# Characteristics of Adults Aged ≥18 Years Evaluated for Substance Use and Treatment Planning — United States, 2019

**DOI:** 10.15585/mmwr.mm7123a1

**Published:** 2022-06-10

**Authors:** Akadia Kacha-Ochana, Christopher M. Jones, Jody L. Green, Christopher Dunphy, Taryn Dailey Govoni, Rebekkah S. Robbins, Gery P. Guy

**Affiliations:** ^1^Office of Strategy and Innovation, National Center for Injury Prevention and Control, CDC; ^2^Office of the Director, National Center for Injury Prevention and Control, CDC; ^3^Inflexxion, Inc., Waltham, Massachusetts; ^4^Division of Overdose Prevention, National Center for Injury Prevention and Control, CDC.

In 2019, 65.8 million U.S. adults reported past-month binge drinking and 35.8 million reported illicit drug use or prescription pain reliever misuse during the past month; 20.4 million met diagnostic criteria for a substance use disorder during the past year (*1*). Approximately 81,000 persons died of a drug overdose* during May 2019–May 2020; excessive alcohol use contributes to an estimated 95,000 deaths per year (*2*). Persons with a substance use disorder are at elevated risk for overdose and associated harms (*3*). To examine the prevalence of past 30-day substance use patterns and the severity of problems experienced across seven biopsychosocial domains (alcohol, drug, employment, family, legal, medical, and psychiatric), CDC used 2019 data from the National Addictions Vigilance Intervention and Prevention Program (NAVIPPRO) Addiction Severity Index-Multimedia Version (ASI-MV) tool (*4*); these data are collected from adults aged ≥18 years who seek substance use treatment in the United States. Alcohol was the most commonly reported substance used during the past 30 days (35.8%), followed by cannabis (24.9%), prescription opioids (misuse) (18.5%), illicit stimulants (14.0%), heroin (10.2%), prescription sedatives or tranquilizers (misuse) (8.5%), cocaine (7.4%), illicit fentanyl (4.9%), and prescription stimulants (misuse) (1.8%).^†^ Polysubstance use (use of two or more substances) during the past 30 days was reported by 32.6% of respondents. Among the biopsychosocial domains measured, 45.4% of assessments reported more severe problems with drugs; others reported psychiatric (35.2%), legal (28.8%), medical (27.4%), employment (25.0%), alcohol (24.2%), and family problems (22.8%). These findings highlight the complex nature of substance use in the United States, the interplay between substance use and mental illness, and the complex challenges that persons with substance use disorder face when seeking treatment. Actions to enhance comprehensive substance use programs that incorporate polysubstance use and co-occurring mental health problems into strategies for prevention, treatment, and response are needed, as is expanded linkage to services. CDC provides data and resources to equip and inform states, territories, and local jurisdictions to help improve opioid prescribing practices, improve linkage to care for the treatment of opioid use disorder, and prevent and reverse overdoses.^§^

NAVIPPRO ASI-MV tool is a validated self-administered, computerized, structured clinical assessment tool administered upon admission to a substance use treatment facility (*5*); the questionnaire is designed to assess each of seven biopsychosocial domains that might affect a respondent’s substance use. A rating is calculated for each domain, indicating the severity of the problem and the need for treatment. The ASI-MV also collects detailed information on lifetime and past 30-day use of tobacco, alcohol, and illicit drugs, as well as use and misuse of prescription drugs.

Using 2019 NAVIPPRO data, CDC assessed the prevalence of past 30-day use overall and by demographic factors (sex, age, race and ethnicity, education, employment status, urban-rural residence, and U.S. Census Bureau region^¶^ of treatment site) for the following substances: alcohol, cannabis, cocaine, illicit stimulants, heroin, illicit fentanyl, prescription opioids (misuse), prescription stimulants (misuse), and prescription sedatives or tranquilizers (misuse). The prevalence of moderate to extremely severe problems** was calculated for each of the seven biopsychosocial domains overall and by demographic characteristics. P-values were calculated using Pearson’s chi-square tests to compare the distribution of demographic characteristics among those who reported past 30-day use of a given substance with those who did not report past 30-day use of that substance; those with a severity score of 4–9 (more severe) in each of the biopsychosocial domains were compared with those with a severity of 0–3 (less severe) in that domain. Respondents with unknown or no response were excluded. P-values <0.05 were considered statistically significant. The prevalences of polysubstance use during the past 30-days and substance combinations were analyzed. All analyses were conducted using SAS (version 7.1; SAS Institute). This activity was reviewed by CDC and was conducted consistent with applicable federal law and CDC policy.^††^

Data from 399 treatment centers in 37 states contributed to the 2019 ASI-MV. Although the centers are primarily substance use treatment centers, other sites, such as driving while intoxicated centers, probation offices, or any site using the ASI-MV tool that agrees to share aggregate assessment data might also be included. Among the 49,138 ASI-MV adults assessed for substance use treatment planning, the majority were men (63.4%), non-Hispanic White persons (65.8%), had a high school education or less (65.4%), and were assessed in metropolitan areas (66.6%) and in the South U.S. Census Bureau region (62.2%) (Table 1).

**TABLE 1:** Prevalence of reported substances used during the past 30 days by adults aged ≥18 years who were assessed for substance use treatment,* by demographic characteristics — United States, 2019

Characteristic	Total assessments, % (N = 49,138)	% Substances used during the past 30 days								
		Alcohol (n = 17,590)	Cannabis (n = 12,222)	Cocaine (n = 3,620)	Illicit stimulants^† ^(n = 6,898)	Heroin (n = 5,020)	Illicit fentanyl (n = 2,421)	Prescription opioid misuse^§ ^(n = 9,073)	Prescription stimulant misuse^¶ ^(n = 888)	Prescription sedatives, tranquilizers, sleeping pills** (n = 4,170)
**Overall**	**100**	**35.8**	**24.9**	**7.4**	**14.0**	**10.2**	**4.9**	**18.5**	**1.8**	**8.5**
**Sex**										
Male	**63.4**	36.5	23.7	7.1	12.0	10.1	4.7	16.1	1.5	6.7
Female	**36.5**	34.6	26.9	7.8	17.5	10.4	5.2	22.6	2.3	11.6
Unknown/No response	**<0.1**	55.0	40.0	10.0	15.0	5.0	10.0	15.0	5.0	15.0
**p-value** ^††^	NA	<0.001	<0.001	0.058	<0.001	0.263	0.437	<0.001	<0.001	<0.001
**Age group, yrs**										
18–24	**14.9**	35.0	34.3	6.0	11.8	8.2	4.4	12.8	1.8	6.7
25–34	**38.0**	33.6	27.5	7.3	16.4	13.3	6.2	21.9	2.1	9.0
35–44	**25.9**	34.8	22.7	7.5	16.2	10.1	5.1	20.1	2.0	9.3
45–54	**13.3**	40.9	18.1	8.2	10.6	7.0	3.3	15.2	1.2	8.2
55–64	**6.8**	43.0	13.8	8.9	6.0	5.0	2.0	13.0	0.8	7.2
≥65	**1.2**	38.2	7.7	4.6	1.9	2.9	1.7	10.1	1.0	5.7
**p-value** ^††^	NA	<0.001	<0.001	<0.001	<0.001	<0.001	0.002	<0.001	<0.001	<0.001
**Race/Ethnicity**										
White, non-Hispanic	**65.8**	35.0	24.3	6.6	16.8	11.7	5.9	21.8	2.3	10.5
Black, non-Hispanic	**13.7**	40.1	26.6	14.3	4.2	7.1	3.3	11.9	0.6	3.1
AI/AN, non-Hispanic	**3.7**	29.4	21.5	2.7	14.3	4.1	1.8	11.4	1.2	4.6
Other,^§§^ non-Hispanic	**4.7**	36.5	31.3	8.0	14.9	9.8	4.3	16.4	1.7	8.1
Hispanic	**12.2**	37.2	24.3	5.1	9.9	7.9	2.5	10.9	1.0	4.8
**p-value** ^††^	NA	<0.001	<0.001	<0.001	<0.001	<0.001	<0.001	<0.001	<0.001	<0.001
**Education level**										
Less than HS	**21.9**	29.6	27.0	9.2	16.4	11.4	5.7	21.3	1.6	7.3
HS diploma	**43.4**	33.1	25.7	7.4	15.7	11.1	5.2	18.8	1.7	8.0
Some college	**24.9**	40.2	24.3	6.8	12.1	9.5	4.7	18.4	2.2	10.0
≥4 yrs college	**9.2**	51.9	18.6	4.8	6.2	5.6	2.5	10.8	2.0	9.7
Unknown/No response	**0.5**	25.2	5.2	2.6	3.0	7.4	3.9	8.3	0.4	0.9
**p-value** ^††^	NA	<0.001	<0.001	<0.001	<0.001	<0.001	0.336	<0.001	0.004	<0.001
**Employment status**										
Full-time	**49.8**	39.4	22.6	5.9	10.3	7.8	3.8	14.1	1.5	6.3
Part-time	**18.9**	35.5	30.0	8.5	16.0	12.4	6.3	22.5	2.5	10.3
Student/Homemaker	**6.0**	34.5	27.2	5.0	16.2	7.1	3.8	22.7	2.6	10.9
Military service	**0.1**	50.8	12.7	0.0	1.6	3.2	1.6	6.3	1.6	0.0
Retired/Disabled	**7.5**	35.9	26.4	11.6	13.5	8.4	4.3	23.0	1.8	12.7
Unemployed	**13.1**	29.9	27.6	11.2	24.1	19.4	8.7	25.5	1.9	12.3
In prison/Hospital	**4.2**	14.8	17.1	4.8	16.9	8.5	3.6	17.4	1.0	4.5
Unknown/No response	**0.5**	26.1	3.4	3.0	3.0	7.7	2.6	14.1	1.7	1.7
**p-value** ^††^	NA	<0.001	<0.001	<0.001	<0.001	<0.001	<0.001	<0.001	<0.001	<0.001
**Urban-rural status^¶¶^**										
Metropolitan	**66.6**	37.5	24.5	8.0	12.7	11.5	5.5	18.3	1.6	8.7
Micropolitan	**20.2**	32.7	25.2	4.5	14.0	5.0	2.6	15.7	1.8	7.3
Rural	**12.9**	31.7	26.3	8.6	21.5	12.2	5.7	24.3	2.8	9.3
Unknown/No response	**0.4**	48.7	15.9	0.5	0.5	0.5	0.0	4.6	1.0	4.1
**p-value** ^††^	NA	<0.001	0.056	<0.001	<0.001	<0.001	<0.001	<0.001	<0.001	<0.001
U.S. Census Bureau region***										
Northeast	**4.2**	42.5	10.6	19.8	3.5	40.4	17.8	17.5	1.2	23.2
Midwest	**17.6**	33.2	29.0	6.6	17.2	8.5	5.0	22.3	2.9	10.1
South	**62.2**	36.2	25.5	7.9	13.6	9.8	4.9	19.3	1.7	7.9
West	**16.0**	35.3	21.5	3.0	15.1	6.0	1.4	11.2	1.0	5.2
**p-value** ^††^	NA	<0.001	<0.001	<0.001	<0.001	<0.001	<0.001	<0.001	<0.001	<0.001

**Abbreviations**: AI/AN = American Indian or Alaska Native; ASI-MV= Addiction Severity Index-Multimedia Version; HS = high school; NA = not applicable; NAVIPPRO = National Addictions Vigilance Intervention and Prevention Program.

* Data were obtained from responses to the NAVIPPRO ASI-MV tool during assessment for substance use at 399 treatment centers located in 37 states.

^†^ Illicit stimulants include illegal amphetamines (e.g., crank, ice, or methamphetamines); this group does not include cocaine.

^§ ^Prescription opioids assessment includes selection of past 30-day misuse of one or more prescription opioid medications. Prescription opioid misuse is any use that is not considered “use as prescribed.” For prescription opioids, “use as prescribed” requires 1) having a current pain problem and taking a prescribed opioid medication for pain during the past 30 days; 2) obtaining the medication only from one’s own prescription; and 3) no use of the medication via an alternate route of administration.

**^¶^** Prescription stimulants assessment includes selection of past 30-day misuse of prescription stimulant medications. Stimulant misuse is any use that is not considered “use as prescribed.” For prescription stimulants, “use as prescribed” is defined as obtaining the stimulant medication only from one’s own prescription and no use of the medication via an alternate route of administration. Misuse is also assigned if a respondent indicates having used the medication during the past 30 days “not in a way prescribed by your doctor to treat a diagnosed attention deficit or hyperactivity disorder.”

** For prescription sedatives and tranquilizers, it was not possible to determine whether these products, which might be obtained by a prescription but are available in the market for a prescription, were misused specifically.

^††^ The p-values represent results of Pearson’s chi-square tests comparing the distribution of demographic characteristics among those assessments who reported past 30-day use of the substance of interest versus those who did not report past 30-day use of that substance. The unknown or no response categories for each demographic characteristic were excluded from the chi-square tests.

^§§^ The Other, non-Hispanic group included those who selected Asian, Native Hawaiian or other Pacific Islander, or “Some other race,” as well as those who selected multiple races. Persons who selected Hispanic ethnicity could be of any race.

^¶¶^ Urban-rural classification of the treatment sites region where assessments were conducted. https://www.cdc.gov/nchs/data_access/urban_rural.htm

*** U.S. Census Bureau region of treatment sites where assessments were conducted are as follows: *Northeast*: Connecticut, Maine, Massachusetts, New Hampshire, New Jersey, New York, Pennsylvania, Rhode Island, and Vermont. *Midwest*: Illinois, Indiana, Iowa, Kansas, Michigan, Minnesota, Missouri, Nebraska, North Dakota, Ohio, South Dakota, and Wisconsin. *South*: Alabama, Arkansas, Delaware, District of Columbia, Florida, Georgia, Kentucky, Louisiana, Maryland, Mississippi, North Carolina, Oklahoma, South Carolina, Tennessee, Texas, Virginia, and West Virginia. *West*: Alaska, Arizona, California, Colorado, Hawaii, Idaho, Montana, Nevada, New Mexico, Oregon, Utah, Washington, and Wyoming. https://www2.census.gov/geo/pdfs/maps-data/maps/reference/us_regdiv.pdf

Alcohol was the substance most commonly reported (35.8%), followed by cannabis (24.9%), prescription opioid misuse (18.5%), illicit stimulants (14.0%), heroin (10.2%), misuse of prescription sedatives or tranquilizers (8.5%), cocaine (7.4%), illicit fentanyl (4.9%), and prescription stimulant misuse (1.8%). Compared with men, women reported higher use of all substances except alcohol. Comparing the prevalence of past 30-day substance use reported in each of the four U.S. Census Bureau regions, the prevalence of heroin, cocaine, illicit fentanyl, and prescription sedative use was highest at Northeast treatment sites, whereas the prevalence of illicit stimulant use was highest at Midwest treatment sites. Among all adults assessed, 32.6% reported use of two or more substances during the past 30 days; the most common polysubstance combinations were alcohol and cannabis (17.2%), followed by cannabis and illicit stimulants (3.7%), and alcohol and prescription opioids (3.4%) (Figure).

**FIGURE:**
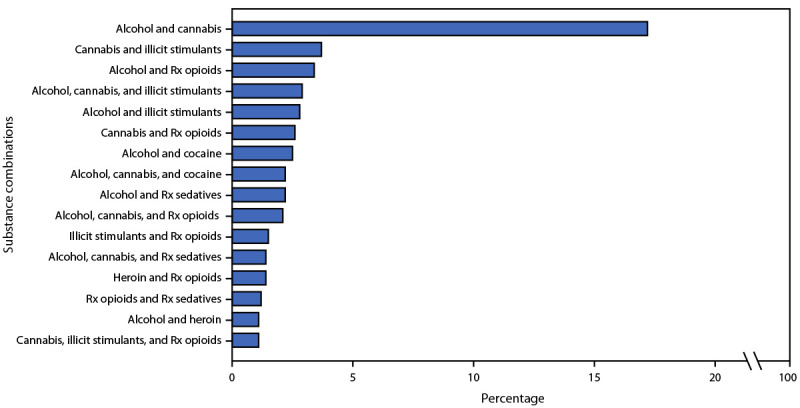
Most common substance combinations reported among past 30-day polysubstance* users aged ≥18 years (N = 16,033) — United States,^†^ 2019 **Source:** National Addictions Vigilance Intervention and Prevention Program, Addiction Severity Index-Multimedia Version tool. **Abbreviations:** ASI-MV = Addiction Severity Index-Multimedia Version; Rx = prescription. * Polysubstance use includes past 30-day use (or prescription medication misuse) of at least two of the following: alcohol, cannabis, heroin, illicit fentanyl, prescription opioids (misuse), barbiturates, prescription sedatives or tranquilizers, cocaine, prescription stimulants (misuse), illicit stimulants (i.e., illegal amphetamines including crank, ice, or methamphetamines; this group does not include cocaine), hallucinogens, inhalants, ecstasy, gamma hydroxybutyrate, ketamine, synthetic cannabinoids (e.g., K2), bath salts, rohypnol, over-the-counter medications, and other unspecified drugs. The remaining unique substance combinations each represented <1% of all combinations among assessments reporting use of two or more substances during the past 30 days. Polysubstance use as displayed in this figure does not necessarily represent use of substances simultaneously. ^†^ Data represent 32.6% of all 2019 adult ASI-MV assessments that reported polysubstance use (i.e., using two or more substances) during the past 30 days.

Among the biopsychosocial domain problems measured, 45.4% of adults assessed reported more severe problems with drugs, followed by psychiatric (35.2%), legal (28.8%), medical (27.4%), employment (25.0%), alcohol (24.2%), and family problems (22.8%) (Table 2). Compared with men, women reported more severe problems for all domains except alcohol. Adults aged 25–34 years reported more severe problems with drugs (49.9%) and those aged 55–64 years reported more severe problems with alcohol (41.1%). Approximately two thirds (67.4%) of unemployed adults assessed experienced more severe drug problems, and retired or disabled adults experienced more severe psychiatric (53.3%) and medical (59.6%) problems.

**TABLE 2:** Percentage of assessments with moderate to extremely severe problems for each of seven biopsychosocial domains among adults aged ≥18 years who were assessed for substance use treatment,* by demographic characteristics — United States, 2019

Characteristics	Total assessments, % (N = 49,138)	% With more severe rating, by domain^†^						
		Medical (n = 13,467)	Employment (n = 2,261)	Legal (n = 14,135)	Family (n = 11,187)	Psychiatric (n = 17,277)	Alcohol (n = 11,877)	Drug (n = 22,289)
**Overall**	**100**	**27.4**	**25.0**	**28.8**	**22.8**	**35.2**	**24.2**	**45.4**
**Sex**								
Male	**63.4**	24.1	22.3	27.1	17.0	27.0	26.1	42.4
Female	**36.5**	33.2	29.5	31.6	32.8	49.3	20.9	50.5
Unknown/No response	**<0.1**	35.0	40.0	30.0	50.0	45.0	30.0	30.0
**p-value** ^§^	NA	<0.001	<0.001	<0.001	<0.001	<0.001	<0.001	<0.001
**Age group, yrs**								
18–24	**14.9**	16.2	23.5	30.5	20.3	31.5	14.6	39.4
25–34	**38.0**	23.3	26.9	31.1	24.1	35.5	19.9	49.9
35–44	**25.9**	29.4	25.9	28.9	24.8	37.3	25.6	47.8
45–54	**13.3**	38.0	23.4	24.6	21.7	36.7	34.9	41.1
55–64	**6.8**	45.1	19.2	21.3	17.4	32.6	41.1	36.0
≥65	**1.2**	38.5	9.9	17.5	11.5	20.2	33.6	22.4
**p-value** ^§^	NA	<0.001	<0.001	<0.001	<0.001	<0.001	<0.001	<0.001
**Race/Ethnicity**								
White, non-Hispanic	**65.8**	28.1	23.8	28.1	24.0	38.0	24.5	48.3
Black, non-Hispanic	**13.7**	29.3	30.3	26.1	19.9	30.2	26.9	43.6
AI/AN, non-Hispanic	**3.7**	26.6	28.6	35.7	21.7	27.4	31.7	40.1
Other,^¶^ non-Hispanic	**4.7**	29.7	26.9	32.4	27.4	40.5	21.8	43.9
Hispanic	**12.2**	21.2	23.4	32.1	18.0	25.8	18.2	33.6
**p-value** ^§^	NA	<0.001	<0.001	<0.001	<0.001	<0.001	<0.001	<0.001
**Education level**								
Less than HS	**21.9**	32.3	33.2	31.3	25.0	37.9	23.0	54.2
HS diploma	**43.4**	26.5	25.4	28.7	21.3	33.7	23.0	48.0
Some college	**24.9**	27.3	21.1	28.2	24.4	37.3	25.5	40.6
≥4 yrs of college	**9.2**	20.9	14.4	25.1	20.5	30.8	29.2	25.9
Unknown/No response	**0.5**	19.1	10.4	15.2	10.9	13.5	16.5	26.1
**p-value** ^§^	NA	<0.001	<0.001	<0.001	<0.001	<0.001	<0.001	<0.001
**Employment status**								
Full-time	**49.8**	18.9	15.1	27.0	17.4	25.7	23.5	35.6
Part-time	**18.9**	30.3	31.4	31.4	28.5	41.5	25.7	53.3
Student/Homemaker	**6.0**	31.0	27.1	31.1	30.8	47.0	19.7	47.2
Military service	**0.1**	17.5	17.5	30.2	14.3	22.2	17.5	17.5
Retired/Disabled	**7.5**	59.6	22.6	24.7	28.1	53.3	31.4	51.1
Unemployed	**13.1**	35.5	50.0	33.3	29.8	47.9	25.5	67.4
In prison/Hospital	**4.2**	28.4	39.3	29.5	18.9	31.7	15.3	47.2
Unknown/No response	**0.5**	24.8	0.0	9.4	11.5	16.2	16.7	20.1
**p-value** ^§^	NA	<0.001	<0.001	<0.001	<0.001	<0.001	<0.001	<0.001
Urban-rural status**								
Metropolitan	**66.6**	25.8	24.8	27.3	20.8	33.0	23.6	43.9
Micropolitan	**20.2**	28.6	22.4	30.3	24.2	38.1	23.6	41.3
Rural	**12.9**	34.3	30.4	34.1	31.1	42.5	28.1	60.4
Unknown/no response	**0.4**	11.3	11.3	32.3	7.2	10.3	22.1	7.2
**p-value** ^§^	NA	<0.001	<0.001	<0.001	<0.001	<0.001	<0.001	<0.001
**U.S. Census Bureau region^††^**								
Northeast	**4.2**	18.6	30.9	18.5	14.9	30.4	49.6	77.9
Midwest	**17.6**	33.9	26.2	28.7	27.3	42.9	27.2	49.0
South	**62.2**	26.9	24.5	29.2	21.8	33.4	20.5	43.9
West	**16.0**	24.7	23.7	29.6	23.8	34.8	28.4	38.5
**p-value** ^§^	NA	<0.001	<0.001	<0.001	<0.001	<0.001	<0.001	<0.001

**Abbreviations:** AI/AN = American Indian or Alaska Native; ASI-MV = Addiction Severity Index-Multimedia Version; HS = high school; NA = not applicable.

* Data were obtained from responses to the National Addictions Vigilance Intervention and Prevention Program ASI-MV tool during assessment for substance use at 399 treatment centers located in 37 states.

^†^ The ASI-MV includes questions for each of seven biopsychosocial domains that might affect a respondent’s substance use; a severity rating is calculated for each domain, indicating the severity of the problem (and the need for treatment). Severity score ratings are calculated via an algorithm that is dependent upon answers to various questions. Interpretation of biopsychosocial domain scores is as follows: 0–1 = no problem; 2–3 = slight problem; 4–5 = moderate problem; 6–7 = severe problem; and 8–9 = extreme problem. For this analysis, scores were combined so that a score falling in the range of 4–9 was considered more severe compared with scores of 0–3.

^§^ The p-values represent results of Pearson’s chi-square tests comparing the distribution of demographic characteristics among persons with a more severe rating indicating a need for treatment (severity score 4–9) to those with a lower domain severity rating indicating that treatment is likely not necessary (severity score 0–3). The unknown or no response categories for each demographic characteristic were excluded from the chi-square tests.

^¶^ The Other, non-Hispanic group included those who selected Asian, Native Hawaiian or other Pacific Islander, or “Some other race,” as well as those who selected multiple races. Persons who selected Hispanic ethnicity could be of any race.

** Urban-rural classification of the treatment sites region where assessments were conducted. https://www.cdc.gov/nchs/data_access/urban_rural.htm

^††^ U.S. Census Bureau region of treatment sites where assessments were conducted are as follows: *Northeast*: Connecticut, Maine, Massachusetts, New Hampshire, New Jersey, New York, Pennsylvania, Rhode Island, and Vermont. *Midwest*: Illinois, Indiana, Iowa, Kansas, Michigan, Minnesota, Missouri, Nebraska, North Dakota, Ohio, South Dakota, and Wisconsin. *South*: Alabama, Arkansas, Delaware, District of Columbia, Florida, Georgia, Kentucky, Louisiana, Maryland, Mississippi, North Carolina, Oklahoma, South Carolina, Tennessee, Texas, Virginia, and West Virginia. *West*: Alaska, Arizona, California, Colorado, Hawaii, Idaho, Montana, Nevada, New Mexico, Oregon, Utah, Washington, and Wyoming. https://www2.census.gov/geo/pdfs/maps-data/maps/reference/us_regdiv.pdf

## Discussion

This study found that among adults assessed for substance use at 399 treatment centers during 2019, alcohol was the most commonly reported substance used during the past 30 days, followed by cannabis, prescription opioid misuse, and illicit stimulants. Nearly one third of all assessments involved polysubstance use, and co-occurring severe problems across multiple biopsychosocial domains were common. Consistent with previous research on substance use patterns in the general population (*1*), men accounted for the majority of assessments for substance use treatment. Women were more likely than men to report use of each of the substances except alcohol; the prevalence of severe problems was higher among women than among men for each of the biopsychosocial domains except alcohol. These patterns might be due to differences in substance use motivation between men and women, how substance use disorders manifest in each sex, barriers to treatment faced by women related to child care and fear of authority involvement (*6*), and differences in the way in which sexes perceive and self-report on biopsychosocial domains.

The observed high rates of polysubstance use among adults assessed for substance use treatment in 2019 are concerning and are consistent with recent drug overdose death data (*7*) and substance use patterns in the general population (*1*). The finding that one third or more of assessments for substance use treatment reported more severe psychiatric problems is also consistent with previous research documenting high rates of mental illness among persons with substance use disorder (*8*). This report focuses on data from 2019, preceding the COVID-19 pandemic; how these trends changed during the pandemic will be the subject of a future report.

Adults assessed in the Northeast U.S. Census Bureau region reported higher past 30-day use of cocaine, heroin, illicit fentanyl, and prescription sedatives, whereas those assessed in the Midwest reported higher past 30-day use of illicit stimulants. The geographic differences in specific substances used during the past 30 days correspond with regional variations in drug overdose deaths (*9*) and the illicit drug supply in the United States (*10*). Continued surveillance of the illicit drug supply and substance use patterns to guide the tailored development of prevention, treatment, and harm reduction interventions will be important when devising public health strategies in U.S. communities.

The findings in this report are subject to at least three limitations. First, ASI-MV data are self-reported and subject to recall and social desirability biases. Second, although ASI-MV collects data from a geographically diverse set of states and treatment programs, it is a convenience sample; therefore, results might not be generalizable to all adults being assessed for substance use treatment.^§§^ Finally, in 2019, 7.4% of ASI-MV assessments were repeat assessments; thus, it is possible for one person to have contributed more than one assessment during 2019.^¶¶^

These findings highlight the complex nature of substance use in the United States, the interplay between substance use and mental illness, and the complex challenges that persons with substance use disorder face when seeking treatment. Actions to enhance comprehensive substance use programs that incorporate polysubstance use and co-occurring mental health problems into strategies for prevention, treatment, and response are needed, as is expanded linkage to services. CDC provides data and resources to equip and inform states, territories, and local jurisdictions to help improve opioid prescribing practices, improve linkage to care for the treatment of opioid use disorder, and prevent and reverse overdoses.

**Summary**

**What is already known about this topic?**

In 2019, 65.8 million U.S. adults reported binge drinking and 35.8 million reported illicit drug use or prescription pain reliever misuse during the past month. Persons with substance use disorders are at high risk for overdose and other harms.

**What is added by this report?**

Among U.S. adults assessed for substance use treatment in 2019, past 30-day use of alcohol (35.8%) and multiple substances (32.6%) were most commonly reported, along with severe problems (e.g., psychiatric, medical, or family) across multiple biopsychosocial domains.

**What are the implications for public health practice?**

Actions to enhance comprehensive substance use programs that incorporate polysubstance use and co-occurring mental health problems into strategies for prevention, treatment, and response are needed, as is expanded linkage to services.

## Footnotes

*
https://emergency.cdc.gov/han/2020/han00438.asp

† Substances assessed in the ASI-MV tool include tobacco, alcohol, cannabis, cocaine, illicit stimulants (i.e., illegal amphetamines including crank, ice, or methamphetamines; this group does not include cocaine), heroin, illicit fentanyl, prescription opioids (misuse), prescription stimulants (misuse), prescription sedatives or tranquilizers, barbiturates, hallucinogens, inhalants, ecstasy, gamma hydroxybutyrate, ketamine, synthetic cannabinoids (e.g., K2), bath salts, rohypnol, over-the-counter medications, and other (unspecified) drugs. Prescription opioid misuse is any use that is not considered “use as prescribed,” which requires 1) having a current pain problem and taking a prescribed opioid medication for pain during the past 30 days; 2) obtaining the medication only from one’s own prescription; and 3) no use of the medication via an alternate route of administration. Prescription stimulant misuse is any use that is not considered “use as prescribed,” which requires obtaining the stimulant medication only from one’s own prescription and no use of the medication via an alternate route of administration. Misuse is also assigned if a respondent indicates having used the medication during the past 30 days “not in a way prescribed by your doctor to treat a diagnosed attention deficit or hyperactivity disorder.” For prescription sedatives and tranquilizers, investigators were unable to determine whether these products, which might or might not be obtained by a prescription but are available in the market with a prescription, were misused specifically.

§ https://www.cdc.gov/drugoverdose/strategies/index.html

¶ U.S. Census Bureau regions: *Northeast*: Connecticut, Maine, Massachusetts, New Hampshire, New Jersey, New York, Pennsylvania, Rhode Island, and Vermont. *Midwest*: Illinois, Indiana, Iowa, Kansas, Michigan, Minnesota, Missouri, Nebraska, North Dakota, Ohio, South Dakota, and Wisconsin. *South*: Alabama, Arkansas, Delaware, District of Columbia, Florida, Georgia, Kentucky, Louisiana, Maryland, Mississippi, North Carolina, Oklahoma, South Carolina, Tennessee, Texas, Virginia, and West Virginia. *West*: Alaska, Arizona, California, Colorado, Hawaii, Idaho, Montana, Nevada, New Mexico, Oregon, Utah, Washington, and Wyoming. https://www2.census.gov/geo/pdfs/maps-data/maps/reference/us_regdiv.pdf

** Severity score ratings are calculated via an algorithm that is dependent upon answers to various questions presented in the ASI-MV. Interpretation of the biopsychosocial domains are as follows: 0–1 = no problem; 2–3 = slight problem; 4–5 = moderate problem; 6–7 = severe problem; and 8–9 = extreme problem. For this analysis, scores were combined so that scores falling in the range of 4–9 were considered a moderately to extremely severe problem and were compared with scores of 0–3.

†† 45 C.F.R. part 46, 21 C.F.R. part 56; 42 U.S.C. Sect. 241(d); 5 U.S.C. Sect. 552a; 44 U.S.C. Sect. 3501 et seq.

§§ Geographic and site participation in NAVIPPRO changes over time, and the network is not formally designed to be nationally representative.

¶¶ In 2019, 7.4% of ASI-MV assessments were repeat assessments, meaning they were completed by a person (represented by a unique identifier) who had already completed one assessment that year.
